# Thalassemia Intermedia and Acute Lymphoblastic Leukemia: Is it a Coincidental Double Diagnosis?

**DOI:** 10.4274/tjh.2014.0068

**Published:** 2014-09-05

**Authors:** Deniz Tuğcu, Zeynep Karakaş, Müge Gökçe, Leyla Ağaoğlu, Ayşegül Ünüvar, Ebru Sarıbeyoğlu, Arzu Akçay, Ömer Devecioğlu

**Affiliations:** 1 Kanuni Sultan Süleyman Education and Research Hospital, Clinic of Pediatric Haematology-Oncology, İstanbul, Turkey; 2 İstanbul University, İstanbul Faculty of Medicine, Department of Pediatric Haematology-Oncology, İstanbul, Turkey

**Keywords:** Thalassemia intermedia, Leukemia, Cancer

## TO THE EDITOR

Beta-thalassemia is not a rare disease in Turkey [1]. ‘Thalassemia intermedia’ describes the patients whose clinical phenotype is placed between thalassemia major and the thalassemia trait despite having homozygote beta-gene mutation. These patients are generally identified with mild-to-moderate anemia later in life than the patients with thalassemia major [2].

A 12-year-old boy presented with headache, pallor, and abdominal distension. Physical examination yielded cervical and inguinal lymphadenopathies, ecchymoses in the lower extremities, and prominent hepatosplenomegaly. Complete blood count revealed hyperleukocytosis (210x109/L), microcytic anemia (Hb: 7.2 g/dL, MCV: 66.9 fL), thrombocytopenia (30x109/L), and mild reticulocytosis (4.3%). Peripheral blood smear showed normoblasts (14%) with 60% blasts. The Coombs test was negative. Viral serology including EBV, CMV, and HIV was negative. Serum ferritin level was 1210 ng/mL. Bone marrow aspiration confirmed the diagnosis of acute lymphoblastic leukemia (ALL) of French-American-British L2 type (90% blasts) and common ALL antigen (+) B ALL was seen with flow cytometry (CD10: 75%, CD19: 86%, CD22: 75%, TdT: 44%). Cytogenetic evaluation revealed 46;XY. Polymerase chain reaction showed negative t(9;22), t(4;11), t(1;19), and t(12;21). No lymphoblasts were demonstrated in the cerebrospinal fluid. He was enrolled in the medium-risk arm of ALL chemotherapy protocol.

From his medical history it was learned that he had a diagnosis of anemia at 5 years of age. Hemoglobin electrophoresis in our clinic showed HbF of 82%, HbA2 of 4%, and HbA of 14%. He had never been transfused to date. Beta-gene analysis revealed homozygote IVS-I-6 (T-C) mutation, pointing to thalassemia intermedia [1].

Chemotherapy was started according to the Children’s Cancer Group. He achieved remission at the end of the phase 1.

The coexistence of thalassemia with cancers such as Hodgkin disease, lymphoma, seminoma, and leukemia has been reported [3,4,5,6,7]. This coexistence could be explained by either genetic or environmental interactions, or it might be thought of as just a coincidence.

Panich et al. reported that the incidence of malignancies in patients with thalassemia was up to 9.4% [8]. Zurlo et al. also reported the death of 8 thalassemia major patients due to malignancy [9]. Such reports suggest the possible association between thalassemia and malignancy. Iron burden, continuous oxidative damage, and viral infections due to transfusions might play roles in the development of malignancy [10].

Whether the above theories hold true or not, one should suspect the possibility of malignancy in patients with thalassemia who develop suggestive signs and symptoms including worsening anemia and splenomegaly. We did not find any relationship between iron overload and cancer in this case. This child was not transfused and did not have evidence of iron overload. Additionally, there were no infections that might have been linked to cancer in this patient. Further studies are needed in order to identify the association between thalassemia and malignancy.

Whether all the above theories hold true or not, one should suspect the possibility of malignancy in patients with thalassemia who develop suggestive signs and symptoms including worsening anemia and splenomegaly. We did not find any relationship between iron overload and cancer in this case. This child was not transfused and did not have evidence of iron overload. Also, there were no any infections that might have been linked to cancer in this patient. Further studies are needed in order to identify the association between thalassemia and malignancy.

## CONFLICT OF INTEREST STATEMENT

The authors of this paper have no conflicts of interest, including specific financial interests, relationships, and/ or affiliations relevant to the subject matter or materials included.
